# A Weighted Gene Co-expression Network Analysis Reveals lncRNA Abnormalities in the Peripheral Blood Associated With Ultra-High-Risk for Psychosis

**DOI:** 10.3389/fpsyt.2020.580307

**Published:** 2020-12-15

**Authors:** Yan Ren, Wei Li, Sha Liu, Zhi Li, Jiaying Wang, Hong Yang, Yong Xu

**Affiliations:** ^1^Department of Psychiatry, Shanxi Bethune Hospital, Taiyuan, China; ^2^Shanxi Academy of Medical Science, Taiyuan, China; ^3^Shanxi Key Laboratory of Artificial Intelligence Assisted Diagnosis and Treatment for Mental Disorder, First Hospital of Shanxi Medical University, Taiyuan, China; ^4^Department of Psychiatry, First Hospital/First Clinical Medical College of Shanxi Medical University, Taiyuan, China; ^5^Department of Hematology, Taiyuan Central Hospital of Shanxi Medical University, Taiyuan, China; ^6^Department of Oncology, The Second Hospital of Shanxi Medical University, Taiyuan, China

**Keywords:** ultra-high-risk for psychosis, long non-coding RNA, mRNA, weighted gene co-expression network analysis, inflammation

## Abstract

**Objective:** The primary study aim was to identify long non-coding RNA (lncRNA) abnormalities associated with ultra-high-risk (UHR) for psychosis based on a weighted gene co-expression network analysis.

**Methods:** UHR patients were screened by the structured interview for prodromal syndromes (SIPS). We performed a WGCNA analysis on lncRNA and mRNA microarray profiles generated from the peripheral blood samples in 14 treatment-seeking patients with UHR who never received psychiatric medication and 18 demographically matched typically developing controls. Gene Ontology (GO) analysis and canonical correlation analysis were then applied to reveal functions and correlation between lncRNAs and mRNAs.

**Results:** The lncRNAs were organized into co-expressed modules by WGCNA, two modules of which were strongly associated with UHR. The mRNA networks were constructed and two disease-associated mRNA modules were identified. A functional enrichment analysis showed that mRNAs were highly enriched for immune regulation and inflammation. Moreover, a significant correlation between lncRNAs and mRNAs were verified by a canonical correlation analysis.

**Conclusion:** We identified novel lncRNA modules related to UHR. These results contribute to our understanding of the molecular basis of UHR from the perspective of systems biology and provide a theoretical basis for early intervention in the assumed development of schizophrenia.

## Introduction

Schizophrenia (SZ) is a serious psychotic disorder caused by a combination of genetic and environmental factors, resulting in functional impairment (1). In the last two decades, researchers have devoted considerable attention to the prodromal state of psychosis in order to offer early intervention (2, 3).This prodromal stage has been termed as “ultra-high-risk, UHR,” “at-risk mental state, ARMS,” or “clinical high risk, CHR” (4). The most prevalent among them are the ultra-high-risk (UHR), which consisted of the presence of attenuated positive symptoms, and/or brief limited intermittent psychotic symptoms, and/or genetic vulnerability along with a significant functional decline (5). About 22–29% of UHR individuals will transition to psychosis, with most transitions occurring in the first 3 years after diagnosis (6). Although UHR is related to both genetic and environmental factors (7), the precise regulatory mechanisms are unknown.

Long non-coding RNAs (lncRNAs) are defined as transcripts longer than 200 nucleotides, which do not encode for proteins (8). A recent study has shown that lncRNAs may contain increasingly complex regulatory information, with roles in transcriptional and post-transcriptional gene silencing (9). LncRNAs might have regulatory functions in diverse biological processes *via* the assembly of distinct regulatory components (10, 11). LncRNAs are highly expressed in the brain and have been found to be dysregulated in many neurological and psychiatric disorders including schizophrenia (12, 13), but little is known about the roles of lncRNAs in UHR.

Brain tissue is ideal for inferring prodromal psychosis, but human brain tissue is rarely available, and previous studies have shown that peripheral blood cells share over 80% of the transcriptome with brain tissues (14). Since peripheral blood has been reported to be viable for providing a “neurological footprint,” blood is therefore a convenient surrogate in UHR (15).

Weighted gene co-expression network analysis (WGCNA) is a systematic method for the analysis of complex gene regulatory networks. Based on microarray data, WGCNA can be used to construct correlation modules and identify candidate biomarkers in complex diseases (16). For instance, a few of hub genes associated with the schizophrenia have been obtained by using this method (17). In our previous study, using WGCNA, we have elucidated two convergent lncRNA modules in patients with early-onset schizophrenia (18), while an integrated analysis of lncRNAs in UHR patients has yet to be explored.

We hypothesized that an integrated WGCNA analysis of lncRNAs would provide novel insight into understanding the pathogenesis of UHR for psychosis. To test this, we performed a co-expression network analysis of blood-based lncRNA and mRNA microarray profiles in 14 patients with UHR who had never received medical treatment and 18 demographically matched typically developing controls (TDCs). Our results identify lncRNA modules associated with UHR for psychosis that has not been described previously. The findings also improve the current understanding of the pathophysiological mechanisms that accompany with UHR.

## Materials and Methods

### Subjects

UHR individuals who had never received psychiatric medication were recruited from the Department of Psychiatry at the First Hospital of Shanxi Medical University. Patients received a face-to-face evaluation by the structured interview for psychosis-risk syndromes (SIPS). The following three diagnostic criteria were evaluated: (a) brief limited intermittent psychotic symptoms (BLIPS), (b) presence of the attenuated positive symptoms state (APSS) (19), and (c) genetic risk and deterioration syndrome (GRDS) with a recent deterioration in general functioning. Prodromal symptoms were defined as the presence of at least one of the three criteria. Exclusion criteria for UHR individuals were a history with a psychotic episode of more than 1 week of duration; serious developmental disorder; history of neurological illness; head trauma; substance abuse; and IQ < 70.

Eighteen gender- and age-matched healthy individuals were recruited as TDCs from neighboring communities. None of these individuals had any major physical disease, neurological disease, mental disease, head trauma, family history of any neurological or psychiatric diseases, or IQ < 70.

This study was approved by the Medical Research Ethics Committee of the First Hospital of Shanxi Medical University. All participants or their legal guardians gave written informed consent. Table 1 shows the demographic characteristics of two groups.

**Table 1:** Demographic properties of UHR individuals and TDCs.

	**UHR (*n* = 14)**	**TDCs (*n* = 18)**	***t*/χ^2^**	***P-*value**
Age, years	16.14 ± 1.406	15.67 ± 2.401	0.658	0.516
Gender (M/F)	9/5	10/8	0.653	0.419

*UHR, ultra-high risk; TDC, typically developing control*.

### RNA Isolation

Peripheral blood samples were collected from all participants. The total RNA was extracted from peripheral blood using the TRIzol reagent (Invitrogen, Carlsbad, CA, USA) and stored at 80°C. Total RNA was quantified with the NanoDrop ND-1000 spectrophotometer (Wilmington, DE, USA). Each sample was evaluated for integrity by agarose gel electrophoresis.

### LncRNA Microarray

The lncRNAs and mRNA sequences were detected using the Arraystar Human 8 × 60 K LncRNA Microarray v2.0 Detection Chip. The array contains 33,045 lncRNA detection probes and 30,215 mRNA detection probes, all from authoritative data sources and the literature. The procedures described in the Agilent One-Color Microarray-based Gene Expression Analysis (Santa Clara, CA, USA) manual were strictly followed. Fluorescent cDNA was purified using the RNeasy Mini Kit (Qiagen, Hilden, Germany). Samples were washed, fixed, and scanned using an Agilent DNA Microarray Scanner (part number G2505C). Experiments were completed by Kang Chen Bio-Tech (Shanghai, China).

### Microarray Data Pre-processing

Chip images were obtained using Agilent Feature Extraction Software (Version 11.0.1.1), and data were processed using GeneSpring GX v11.5.1 (Agilent Technologies). Raw expression data were transformed by log2, background corrected and normalized by quantile normalization for both lncRNA and mRNA data sets. In total, 21,250 lncRNAs and 21,654 mRNAs were obtained for subsequent analyses.

### Weighted Gene Co-expression Network Analysis (WGCNA) and Gene Ontology Enrichment Analysis

We used the WGCNA package to build a weighted gene co-expression network. First of all, differentially expressed genes (DEGs) screening was applied to selected the most variable transcripts (20). Transcripts with logFC > (abs(log2(1.8))) and *P*–value < 0.05 were selected for subsequent analysis. In total, the 4,105 lncRNAs and 4,013 mRNAs were selected for construction of co-expression networks, respectively. Network preservation was assessed and the preservation statistic Z_summary_ >2.0 indicates that the module is significantly preserved (21). The Pearson's correlation matrices were calculated for all pairs of RNAs, and then transformed into adjacency matrices using a power function amn = power (Smn, β) = |Smn|β. The parameter, β, was optimized to maintain both the scale-free network and sufficient node connectivity. Here, the power of β = 16 for lncRNA and β = 18 for mRNA were chosen. A dynamic tree cut algorithm was used to identify gene co-expression modules, built with the default value of SplitDepth for robust module detection in WGCNA (cutHeight = 200; minModuleSize = 30; deepSplit = 2; mergeCutHeight = 0.15). Modules were defined as the branches cutoff of the tree and each module was labeled in unique colors. The module eigengene (ME) was defined as the first principal component of the module. Modules which were most relevant to the disease were then identified using WGCNA package. RNAs that tended to have the highest connectivity in the selected module (|GS| > 0.9 and |dat KME| > 0.85) were extracted and defined as hub RNAs. Gene Ontology (GO) analysis was applied to reveal functions of gene products by the “GOenrichmentAnalysis” function in the WGCNA package. The *p*-value < 0.05 was used as the cut-off criterion.

### Canonical Correlation Analysis (CCA)

The R package CCP (Significance Tests for Canonical Correlation Analysis) was used for a CCA of hub lncRNAs and hub mRNAs (22), as well as to explore the correlations between these multivariate variables (23).

## Results

### LncRNA Co-expression Networks

**Figure 1:**
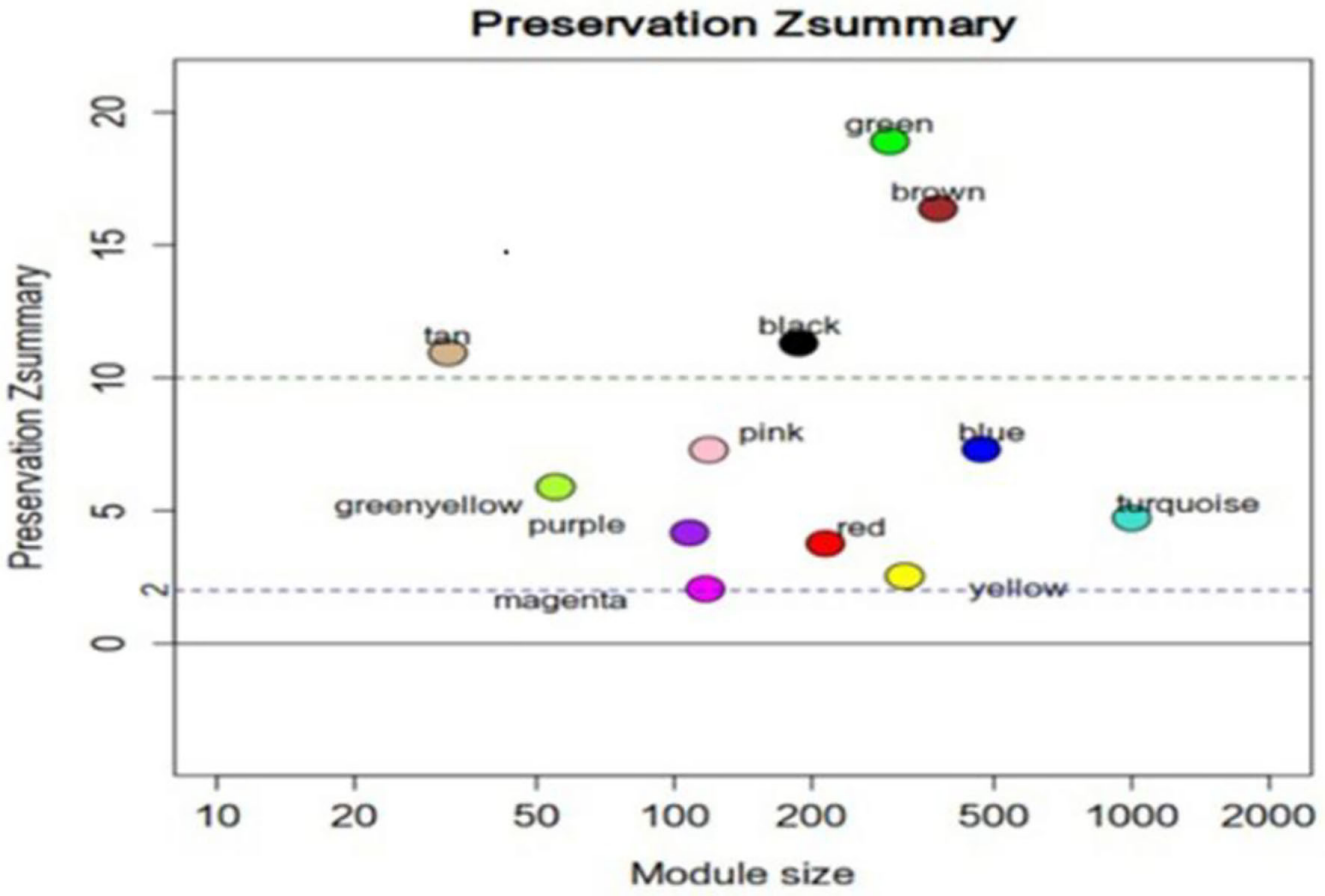
Z_summary_ statistics for the preservation of lncRNA modules. Most lncRNA network modules from individuals at ultra-high risk (UHR) for psychosis or typically developing controls(TDCs) are well-preserved. Z_summary_ is the summary statistics of module preservation. The vertical axis represents the Z_summary_ score, and the horizontal axis represents the LncRNA numbers in each module. Z_summary_ < 2.0 implies no evidence for preservation.

**Figure 2:**
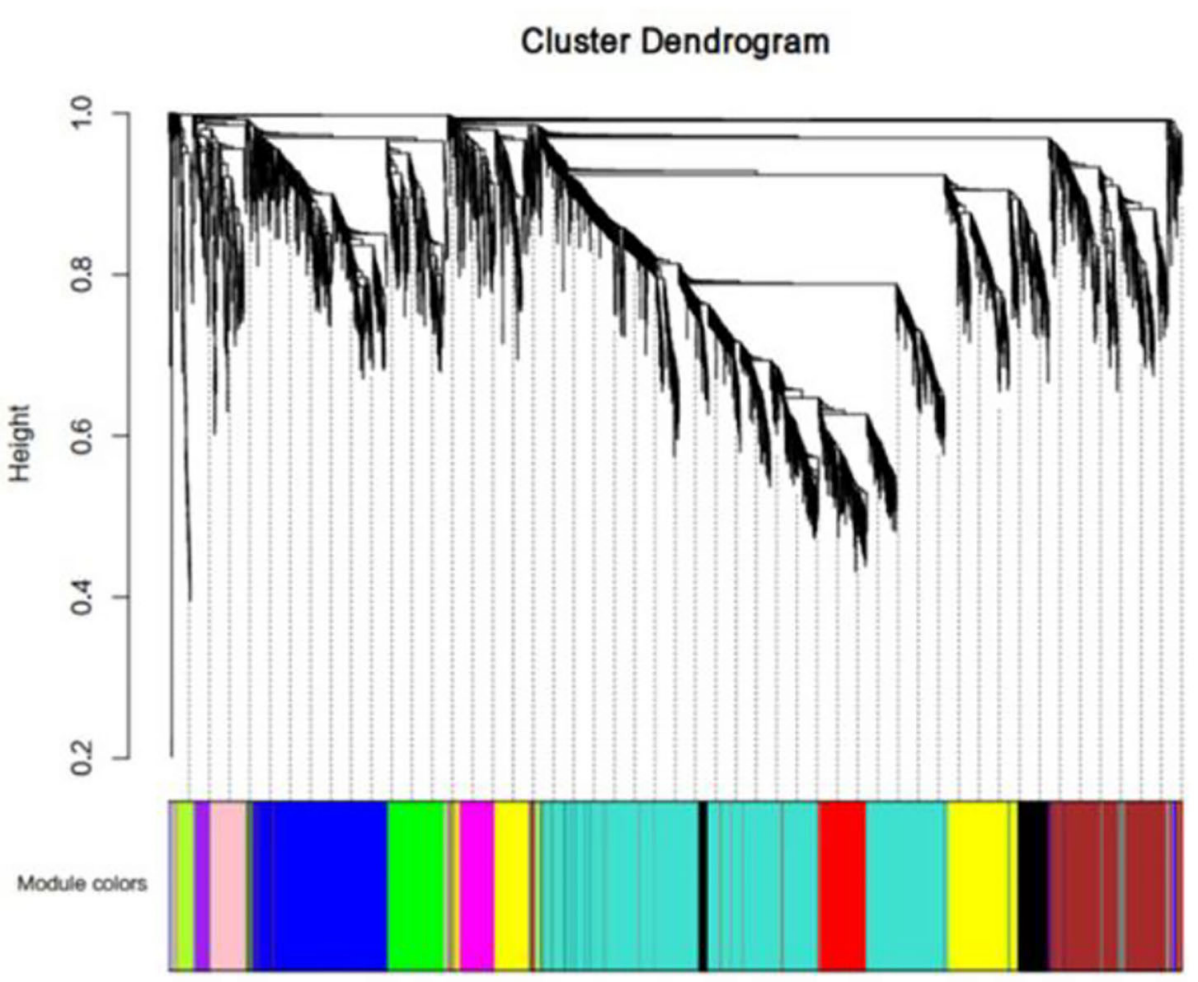
LncRNA co-expression network modules. The lncRNAs were organized into twelve co-expression network modules. Different colors represent different modules, and gray represents lncRNAs that cannot be merged into any module.

**Figure 3:**
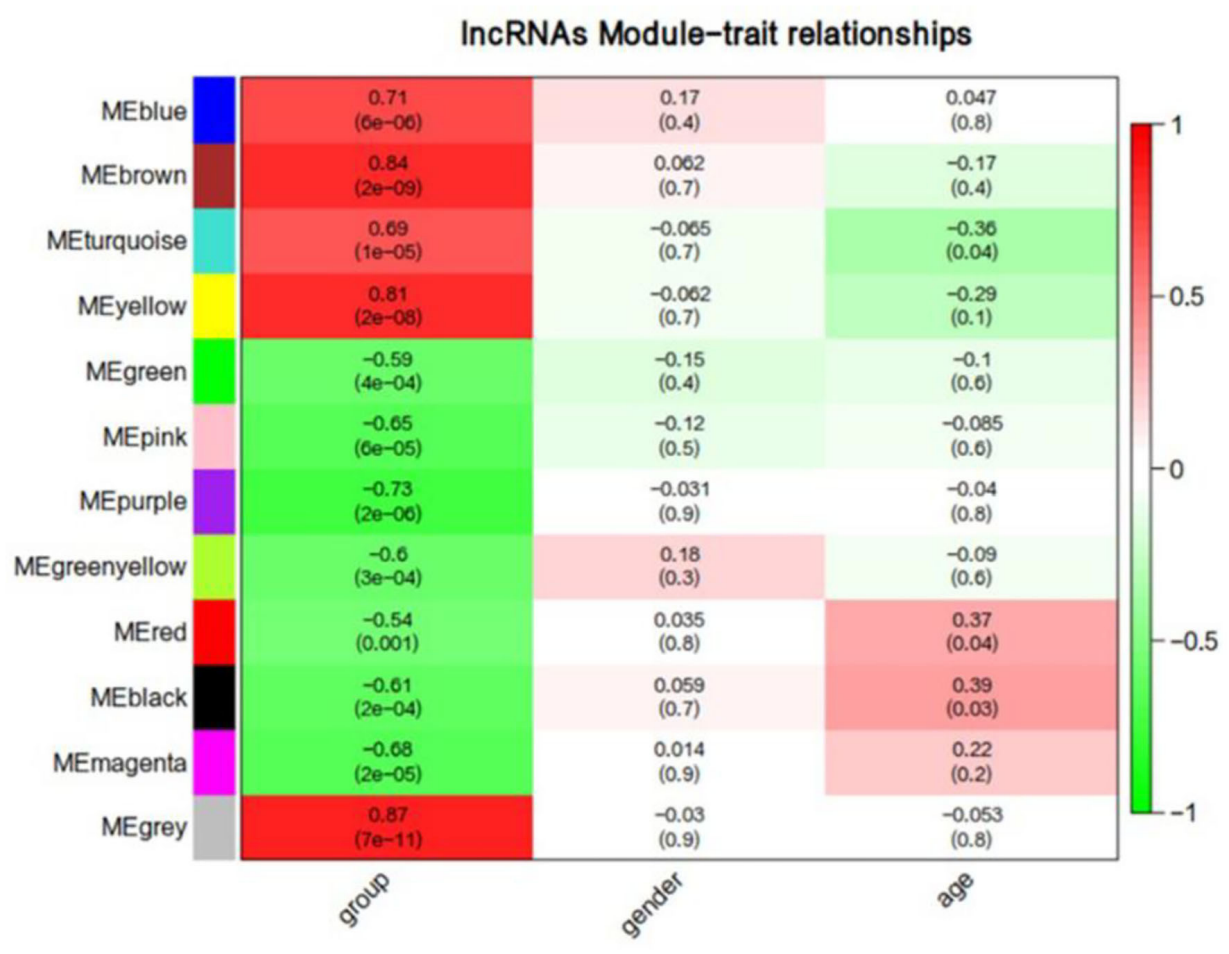
Correlation between lncRNAs module eigengenes (ME) disease characteristics. Each row corresponds to a module eigengene, each column to a trait. Each cell contains the corresponding correlation and the *P*-value. The table is color-coded by the correlation according to the color legend. Group represents the disease trait of individuals at ultra-high risk (UHR) for psychosis or typically developing controls.

**Figure 4:**
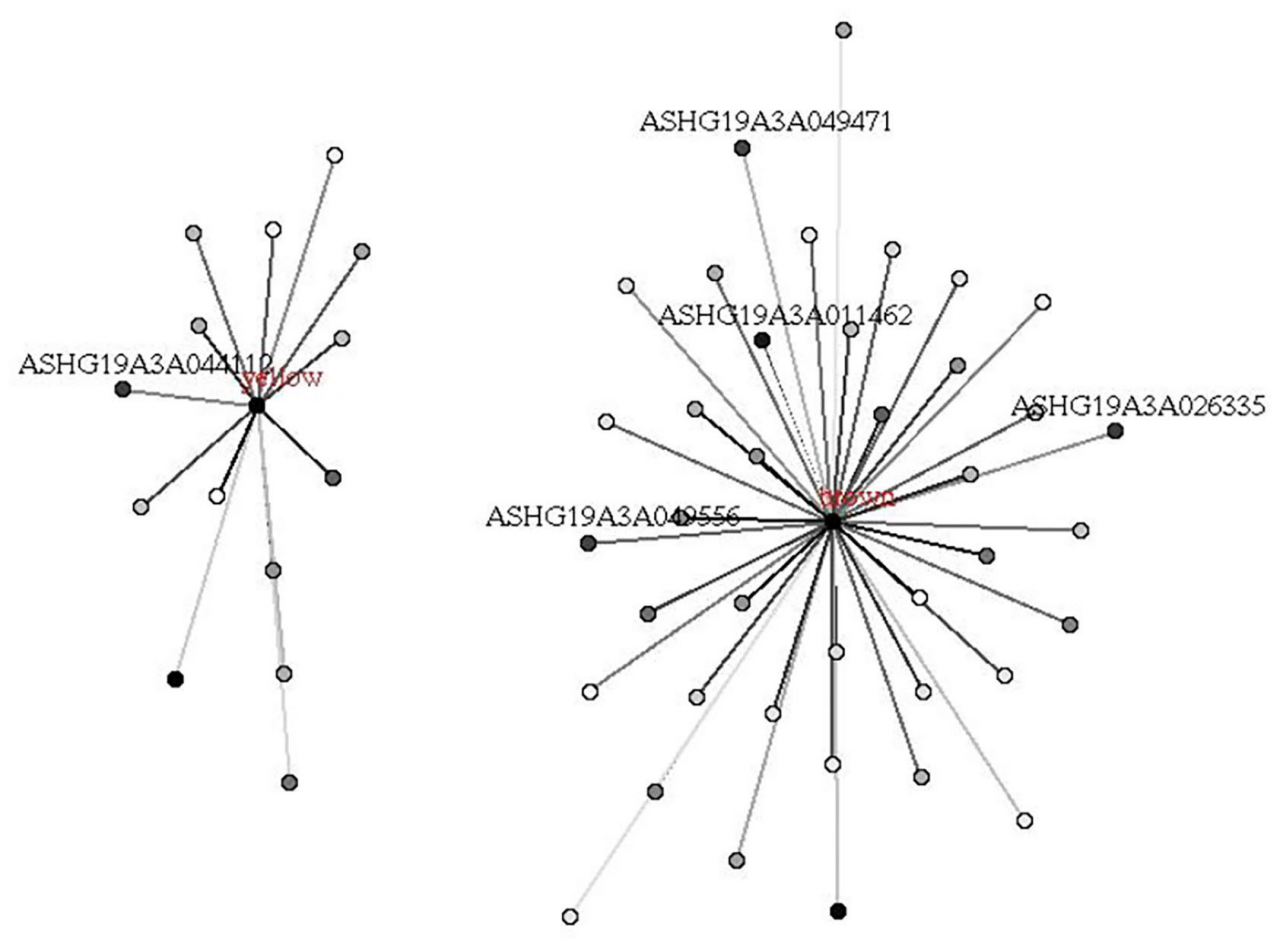
Hub lncRNAs in brown and yellow modules related to UHR. Hub lncRNAs in the module were identified according to the absolute values of GS (gene significance) and kME (eigengene connectivity) of each lncRNA. The depth of point represents the absolute value of GS, and the depth of line represents the absolute value of kME.

We first performed a WGCNA to construct co-expression networks for UHR individuals and TDCs samples separately. As determined by the Z_summary_ statistic, the modules in the two groups were well-preserved and had no global perturbations between two groups (Figure 1). Subsequently, we combined the two data sets to build the network and identify modules, as previously described in gene co-expression networks in the brain (24). The lncRNAs with similar patterns of expression were grouped into modules and a total of 11 modules (Figure 2) were identified. Each lncRNA module eigengene (Supplementary Table 1) was used to identify the correlations between modules and phenotypes which included the disease, age and gender. The modules that were most significantly related to disease were the brown and yellow modules (Figure 3). The brown module contained 526 lncRNAs, with four hub lncRNAs identified (Figure 4). Among which ASHG19A3A011462 and ASHG19A3A026335 were up-regulated in UHR individuals, while ASHG19A3A049471 and ASHG19A3A049556 were down-regulated. The yellow module contained 473 lncRNAs, with one hub lncRNA (ASHG19A3A044112) identified (Figure 4), which was down-regulated in UHR individuals. However, the function of the identified lncRNAs remains unknown.

### mRNA Co-expression Networks and Functional Annotations

**Figure 5:**
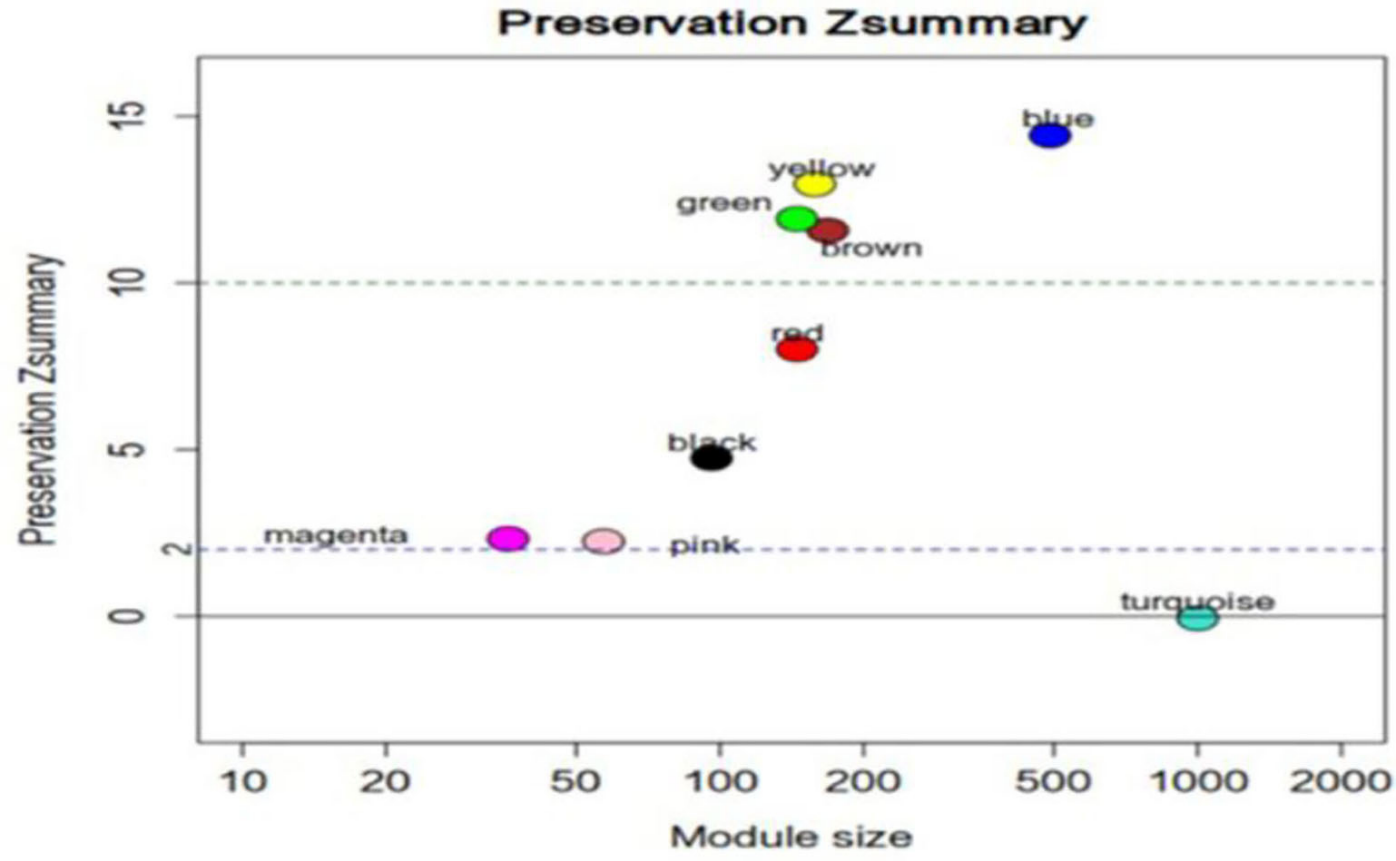
Z_summary_ statistics for the preservation of mRNA modules. Most mRNA network modules from individuals at ultra-high risk (UHR) for psychosis or typically developing controls(TDCs) are well-preserved. Z_summary_ is the summary statistics of module preservation. The vertical axis represents the Z_summary_ score, and the horizontal axis represents the LncRNA numbers in each module. Z_summary_ < 2.0 implies no evidence for preservation.

**Figure 6:**
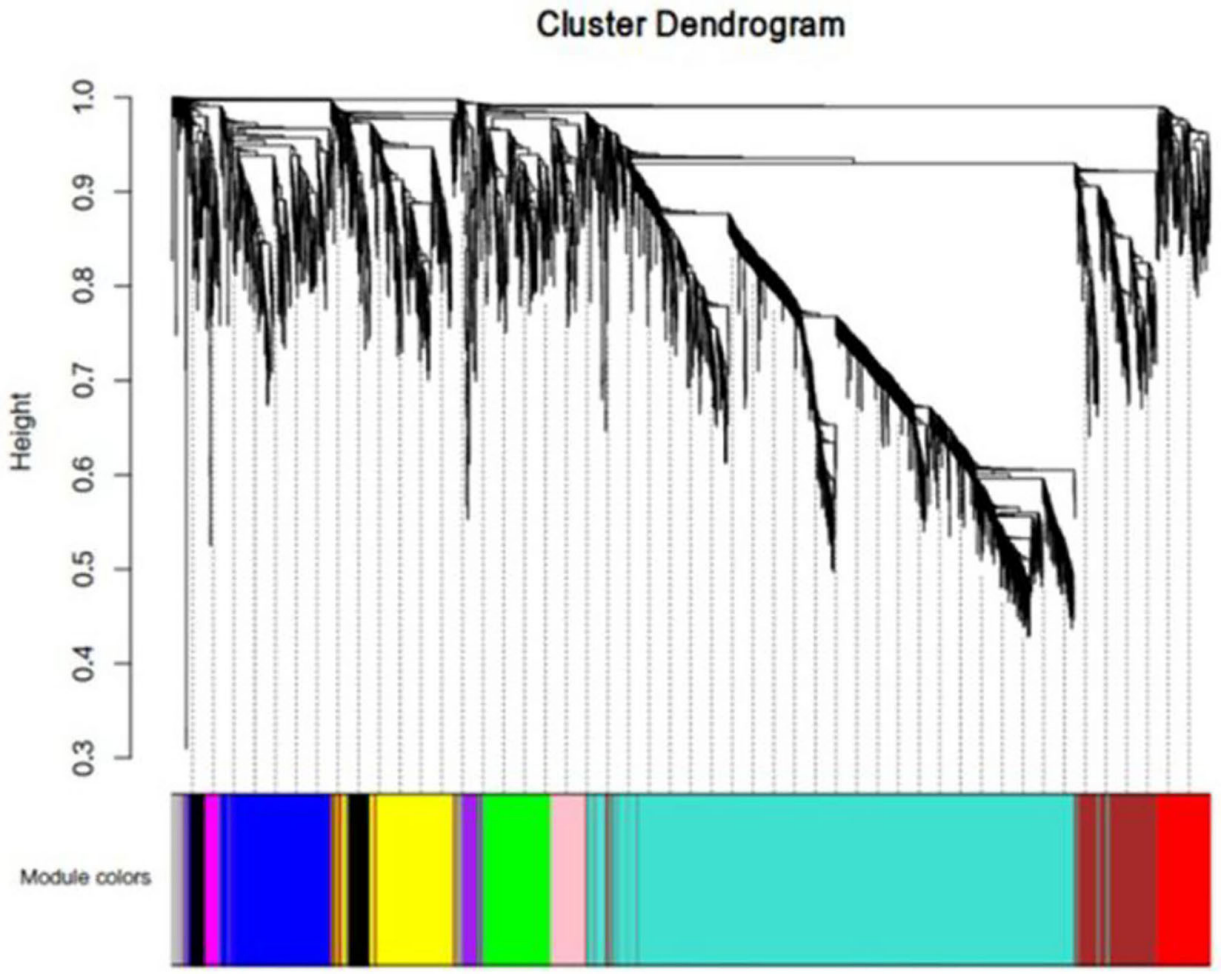
mRNA co-expression network modules. The mRNAs were organized into eleven co-expression network modules. Different colors represent different modules, and gray represents lncRNAs that cannot be merged into any module.

**Figure 7:**
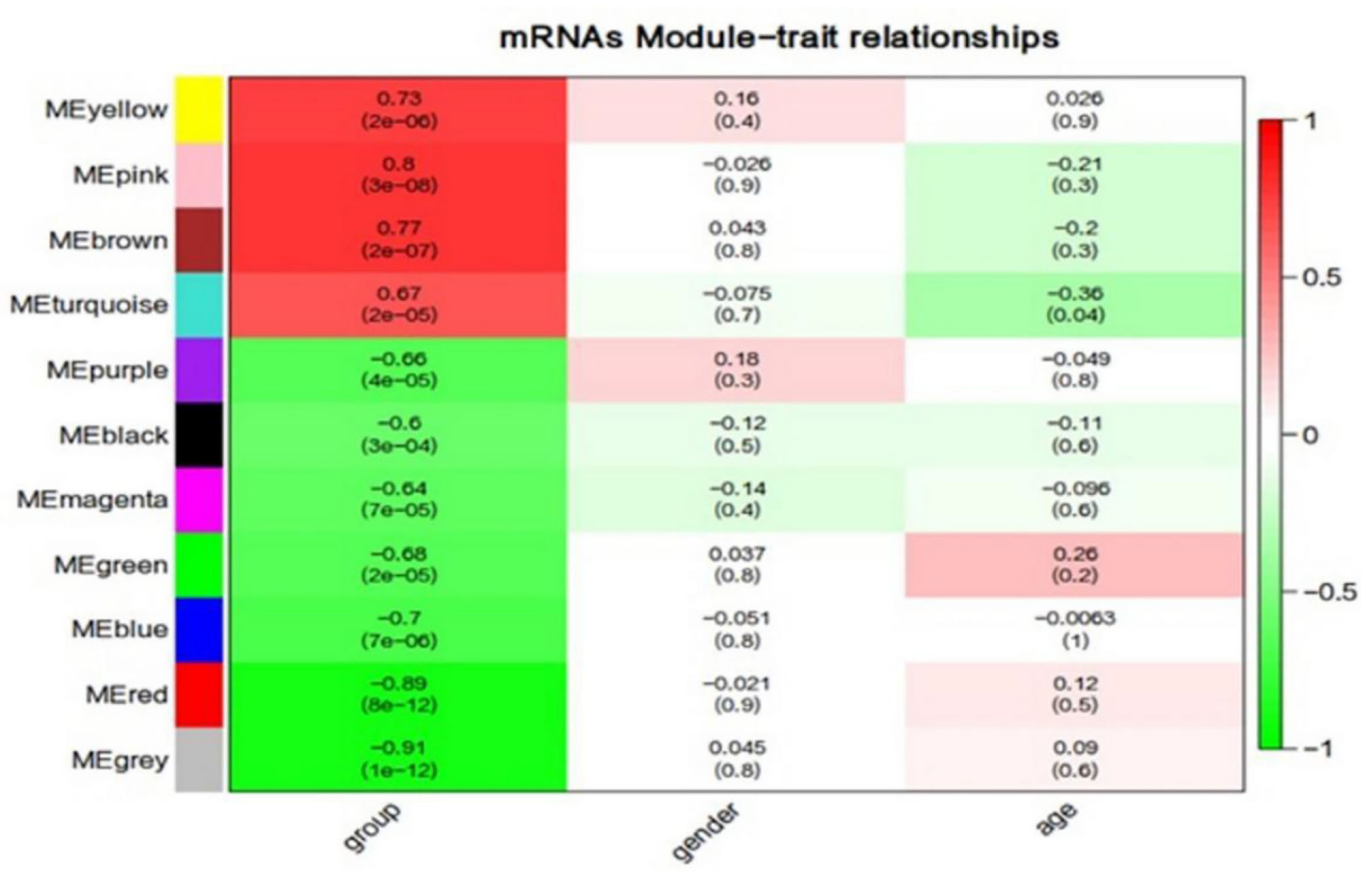
Correlation between mRNAs module eigengenes (ME) disease characteristics. Each row corresponds to a module eigengene, each column to a trait. Each cell contains the corresponding correlation and the *P*-value. The table is color-coded by the correlation according to the color legend. Group represents the disease trait of individuals at ultra-high risk (UHR) for psychosis or typically developing controls.

**Figure 8:**
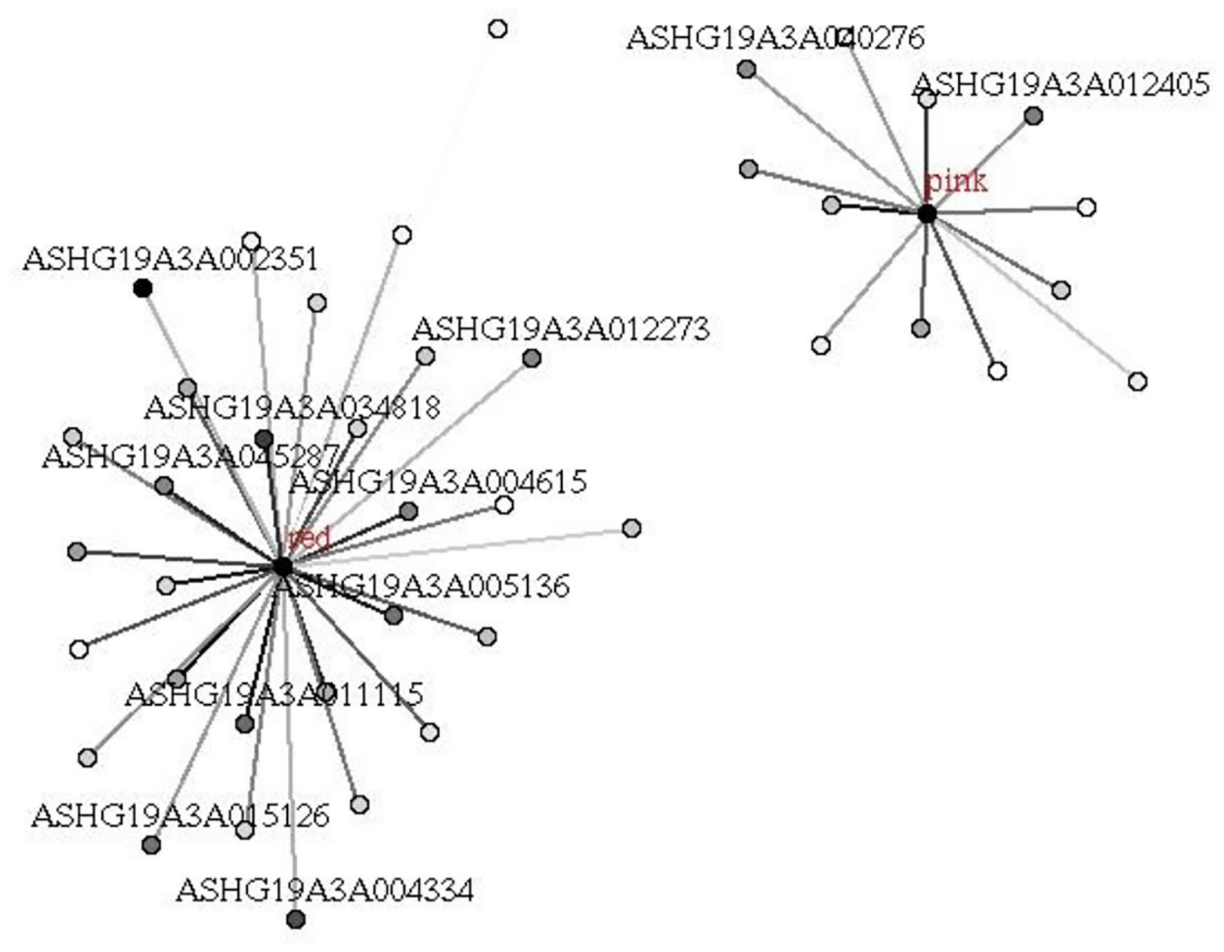
Hub mRNAs in red and pink modules related to UHR. Hub mRNAs in the module were identified according to the absolute values of GS (gene significance) and kME (eigengene connectivity) of each lncRNA. The depth of point represents the absolute value of GS, and the depth of line represents the absolute value of kME.

We used the same method described above to construct mRNA co-expression networks for UHR individuals and TDCs. Z_summary_ statistic showed that the modules were well-preserved in UHR group and TDCs group (Figure 5). By using the combined data set, we identified 10 mRNA modules (Figure 6). The red and pink modules were highly significantly correlated with UHR, shown in Figure 7. The red module contained 208 mRNAs. A GO enrichment analysis showed this module was significantly enriched for inflammation (Supplementary Table 2). We confirmed that there are nine hub mRNAs in the red module, including TOM1, TJP2, RALY, ARRB1, STX10, ASNA1, KCNJ13, GNB2, and STX5, all of which were down-regulated in UHR individuals. The pink module contained 145 mRNAs and two hub mRNAs (i.e., C19orf60 and HLX) were identified, both of which were down-regulated in UHR individuals (Figure 8). A GO enrichment analysis showed that this module was significantly enriched for the immune response (Supplementary Table 2).

### Correlation Between Hub lncRNAs and Hub mRNAs

As shown in Table 2, the first two pairs of canonical variables were statistically significant (*P* < 0.05). We identified specific hub lncRNAs that were highly correlated with hub mRNAs.

**Table 2:** Canonical correlation coefficients for relationships between hub lncRNAs and hub mRNAs.

**Canonical variables**	**Correlation coefficient**	***F***	***P***
V1-U1	0.983	3.782	<0.001
V2-U2	0.843	1.676	0.031
V3-U3	0.731	1.223	0.261
V3-U3	0.575	0.905	0.569
V3-U3	0.466	0.794	0.601

*V1:the first canonical variables of LncRNA, U1:the first canonical variables of mRNA*.

To find out the key factors in each group of canonical variables, we presented the loadings for the canonical function (Supplementary Table 3). Canonical loading presents a correlation between the original variable and its corresponding canonical variate. These values reflect the degree of a variable be represented by a canonical variate. Canonical loadings for variables suggested that LncRNA ASHG19A3A044112 (0.513) and ASHG19A3A049556 (0.489) had more effect to form the variables of lncRNA (V2). And the RALY, ARRB1, ASNA1, C19orf60, GNB2, and STX5 were more important factors to form the second fair for variables of mRNA (U2). These results indicated a high correlation between the lncRNA and mRNA.

## Discussion

In this study, we performed a systematic analysis of lncRNAs co-expression networks in the drug-naïve patients with UHR for psychosis. Importantly, we used the gene co-expression networks constructed from lncRNA expression profile and we found the existence of two lncRNAs modules relevant to UHR. Furthermore, we revealed their potential biological functions based on the functions of corresponding mRNAs with highly correlated expression patterns. These findings provide insight into the pathogenesis of UHR from the perspective of systems biology.

There is abundant evidence linking changes in lncRNA expression to the molecular pathology of central nervous system diseases (25, 26). The epigenetic mechanisms underlying the precursory stage of schizophrenia are largely unknown. Network analysis reveals the higher-order relationships of the transcripts and can greatly alleviate the multiple testing problems (27). In addition, this network analysis reduces potentially confounding factors, such as prior knowledge bias from the published literature or batch effects when constructing the networks from multiple profiling data. Strikingly, compared with single genes, biological modules may represent specific subsets of enriched biological processes.

Our integrative analysis reveals several modules of particular interest. Two lncRNAs modules were identified to be associated with UHR, named brown and yellow modules, respectively. Hubs are topologically central in the module, having maximal informational connections with other nodes. In our study, a total of five lncRNAs were identified as hub LncRNAs in these modules. Two mRNAs modules, named red and pink modules, were highly significantly correlated with UHR. GO enrichment analysis showed these two modules were significantly enriched for immune response and inflammation.

There is growing evidence for the important roles of immune-inflammatory abnormalities in the pathogenesis of UHR for psychosis. Immune-mediated glutamate-dopaminergic dysregulation may lead to various positive symptoms of the prodromal stage due to the early chronic immune system damage (28). Anti-inflammatory treatment can effectively improve symptoms of patients with UHR (29). Inflammation may progress slowly and be difficult to detect at the early stage. Maternal infections trigger early immune changes in fetal development and are immune-related risk factors for schizophrenia (30). Disturbances in the expression of inflammation-related genes may lead to clinical symptoms. As the most important immune inflammatory cells in the brain, the microglia hypothesis of schizophrenia is widely accepted (31). The excessive activation of microglia results in the production of a large number of toxic factors, inhibition of the growth of nerve cells, withering, and promotion of the occurrence and progression of degenerative diseases (32). These results have been confirmed in animal model (33). Increased microglial activity in individuals with UHR and schizophrenia occur at an early stage of the disease (34). Abnormal immune inflammatory responses of the central nervous system may play a key role in the UHR.

Our results should be interpreted with caution due to several limitations. First, the sample size of our current study was relatively small, which may limit the statistic power. Second, some confounding factors in the data analysis should be considered because the potential batch effects and multiple-testing issue from RNA-sequencing data could confound true biological relationships of the data to a certain extent. Another issue is the tissue specificity of gene expression. Here, we measured lncRNA and mRNA expression in peripheral blood but not in the brain. However, between 35 and 80% of known transcripts are present in both brain and blood tissue samples, estimates of cross-tissue correlation in expression levels has been reported to range from 0.25 to 0.64 (35). We are also aware that issues pertaining to blood-brain gene expression correlations are not addressed. The advantage of using blood samples is that it can be acquired without using invasive procedures, and repeated sampling from the same individual is applicable, which facilitates longitudinal studies. Additional studies on larger cohorts of patients as well as brain-blood correlation of the identified genes are needed to further investigate the implication of the pathophysiology of UHR.

In conclusion, at a system level, our research indicated that two convergent lncRNA dysregulation of the expression in peripheral blood was involved in the early pathological processes of UHR that lead to psychosis. The lncRNAs could correlate with mRNAs, and lead to an immune-inflammatory abnormalities in the pathogenesis of UHR for psychosis. We hope that our research can provide a basis for the search for biomarkers for the UHR individuals as well as for the development of strategies for early intervention of UHR.

## Data Availability Statement

The data have been assigned the ArrayExpress database at EMBL-EBI (www.ebi.ac.uk/arrayexpress) under accession number E-MTAB-9804, and will be publicly available on December 1, 2020.

## Ethics Statement

The studies involving human participants were reviewed and approved by Medical Research Ethics Committee of the First Hospital of Shanxi Medical University. Written informed consent to participate in this study was provided by the participants' legal guardian/next of kin.

## Author Contributions

YR and YX designed the study. WL, SL, and YR drafted the manuscript. YR and HY recruited samples. ZL and JW performed the analyses. All authors reviewed and approved the final manuscript.

## Conflict of Interest

The authors declare that the research was conducted in the absence of any commercial or financial relationships that could be construed as a potential conflict of interest.
