# Supplementary material for: A Weighted Gene Co-expression Network Analysis Reveals lncRNA Abnormalities in the Peripheral Blood Associated With Ultra-High-Risk for Psychosis
Source: Front Psychiatry. 2020 Dec 15;11:580307. doi: 10.3389/fpsyt.2020.580307 (PMC7769947; doi:10.3389/fpsyt.2020.580307)
Supplement: Supplementary file 3 [file Table_3.DOC]

Supplementary Table3 Canonical loadings of original variables with their canonical variables

|  |  | **lncRNA set** | | | |  | **mRNA set** | | | | | | | | | | |
| --- | --- | --- | --- | --- | --- | --- | --- | --- | --- | --- | --- | --- | --- | --- | --- | --- | --- |
|  | ASHG19A3A011462 | ASHG19A3A026335 | ASHG19A3A044112 | ASHG19A3A049471 | ASHG19A3A049556 |  | ASHG19A3A002351 | ASHG19A3A004334 | ASHG19A3A004615 | ASHG19A3A005136 | ASHG19A3A011115 | ASHG19A3A012273 | ASHG19A3A012405 | ASHG19A3A015126 | ASHG19A3A034818 | ASHG19A3A040276 | ASHG19A3A045287 |
|  |  |  |  |  |  | ***Gene*** | TOM1 | TJP2 | RALY | ARRB1 | STX10 | ASNA1 | C19orf60 | KCNJ13 | GNB2 | HLX | STX5 |
| ***V1*** | 0.001 | -0.041 | 0.262 | -0.007 | -0.022 | ***U1*** | 0.127 | 0.046 | -0.083 | 0.190 | -0.044 | 0.033 | 0.072 | 0.000 | -0.077 | -0.047 | 0.067 |
| ***V2*** | -0.076 | 0.083 | **0.513** | 0.013 | **0.489** | ***U2*** | -0.302 | 0.124 | **-0.565** | **1.611** | -0.022 | **0.812** | **0.395** | -0.124 | **-0.678** | -0.057 | **-1.066** |

V1,V2:1st and 2nd lncRNA canonical variables; U1,U2:1st and2nd mRNA canonical variables.
